# Cobalt-Doped Porous Carbon Nanosheets Derived from 2D Hypercrosslinked Polymer with CoN_4_ for High Performance Electrochemical Capacitors

**DOI:** 10.3390/polym10121339

**Published:** 2018-12-04

**Authors:** Yuanhai Chen, Fengru Liu, Feng Qiu, Chenbao Lu, Jialing Kang, Doudou Zhao, Sheng Han, Xiaodong Zhuang

**Affiliations:** 1 School of Chemical and Environmental Engineering, Shanghai Institute of Technology, Haiquan Road 100, Shanghai 201418, China; yuanhai_charles@163.com (Y.C.); lfru0719@163.com (F.L.); 176061210@mail.sit.edu.cn (J.K.); 176062121@mail.sit.edu.cn (D.Z.); 2 School of Chemistry, University of Bristol, Cantock’s Close, Bristol BS8 1TS, UK; 3 School of Chemistry and Chemical Engineering, State Key Laboratory of Metal Matrix Composites, Shanghai Jiao Tong University, 800 Dongchuan Road, Shanghai 200240, China; castle@sjtu.edu.cn

**Keywords:** transition metal-doping, porous carbon, hypercrosslinked polymer, two-dimensional nanosheet, supercapacitor

## Abstract

Cobalt-doped graphene-coupled hypercrosslinked polymers (Co-GHCP) have been successfully prepared on a large scale, using an efficient RAFT (Reversible Addition-Fragmentation Chain Transfer Polymerization) emulsion polymerization and nucleophilic substitution reaction with Co (II) porphyrin. The Co-GHCP could be transformed into cobalt-doped porous carbon nanosheets (Co-GPC) through direct pyrolysis treatment. Such a Co-GPC possesses a typical 2D morphology with a high specific surface area of 257.8 m^2^ g^−1^. These intriguing properties of transition metal-doping, high conductivity, and porous structure endow the Co-GPC with great potential applications in energy storage and conversion. Utilized as an electrode material in a supercapacitor, the Co-GPC exhibited a high electrochemical capacitance of 455 F g^−1^ at a specific current of 0.5 A g^−1^. After 2000 charge/discharge cycles, at a current density of 1 A g^−1^, the specific capacitance increased by almost 6.45%, indicating the excellent capacitance and durability of Co-GPC. These results demonstrated that incorporation of metal porphyrin into the framework of a hypercrosslinked polymer is a facile strategy to prepare transition metal-doped porous carbon for energy storage applications.

## 1. Introduction

Over the past decades, supercapacitors have become promising electronic devices for use in meeting future energy-storage requirements in industrial energy management, electric vehicles, and memory back-up systems. This is owing to the supercapacitor’s unique performance, including superior power density, long life cycle, and short charging time [1,2,3]. Carbon-based materials with low electric resistance, such as carbon nanofibers [4,5], activated carbon [6,7], carbon nanotubes [8,9], and carbon aerogel [10,11], have been developed as electrode materials for applications in supercapacitors. Unfortunately, these materials often possess shortcomings, such as low specific surface area, uncontrolled pore size distribution, high cost, and poor mechanical properties, which limit to some degree the materials’ application in supercapacitors. Recently, graphene, featuring a flat monolayer of carbon atoms arranged in a two-dimensional (2D) honeycomb lattice, exhibited intriguing physical and chemical properties, including a high charge mobility, superior thermal conductivity, a large specific surface area, and good chemical stability [12,13,14]. It has also been utilized widely in electrochemical sensors [15], energy storage and conversions [16,17], and field-effect transistors [18,19], amongst others. According to the charge-storage mechanism, all carbon-based materials possess electric double-layer capacitors (EDLC) characterization, and a low specific surface area, which leads to the material’s low capacitance [20]. Therefore, the design and construction of new 2D materials with high conductivity, controllable hierarchical porous structure, and large redox activity is highly desirable for electrochemical capacitors.

To date, many materials have been introduced onto graphene to achieve high performance supercapacitors. Several metal oxides and sulfides (e.g., MnO_2_ [21,22], NiO_x_ [23], CoO_x_ [24], MoS_2_ [25], and Ni_3_S_2_ [26]) show a fast-Faradaic reaction, leading to a higher specific capacitance for these pseudocapacitive electrode materials. Thus, the preparation of graphene-based hybrids through the loading of inorganic materials onto the surface of graphene has been confirmed as a facile strategy to obtain high performance electrode materials [27]. Using the example of Ni_3_S_2_, Zhuang et al. reported the preparation of graphene-coupled flower-like nickel sulfide monoliths using an F127-assisted hydrothermal method and high-temperature pyrolysis. The as-prepared graphene-based hybrid showed ultra-high specific capacitances of up to 1315 F g^−1^, as well as high stability through 1000 charge/discharge cycles at 1 A g^−1^ [26]. However, the severe aggregation tendencies of graphene and the agglomeration of inorganic nanoparticles in the hybrid structure could hinder electron and mass transport [28], resulting in low charge-discharge rates and poor gravimetric capacitances. Compared to that of heteroatom doping [29,30], the transition metal-doping strategy could not only enhance the conductivity and electrochemical stability of the framework structures, but it could also provide several electrochemically active sites, thereby exhibiting great potential for use in energy-related applications [31]. In recent years, transition metal-doped porous carbon has been reported for use in supercapacitor applications. To the best of our knowledge, the development of 2D metal-doped porous carbon with high performance electrochemical capacitors is still a challenge [32].

Based on our previous work [33], cobalt-doped 2D graphene-coupled hypercrosslinked porous polymer nanosheets (Co-GHCP) were constructed for the present work using a nucleophilic substitution reaction of 5,10,15,20-tetra(4-pyridyl) Co (II) porphyrin and graphene-coupled poly (vinylbenzyl chloride-co-divinylbenzene), prepared via RAFT emulsion polymerization with the macromolecular chain-transfer agent of GO-DDAT. Cobalt-doped graphene-coupled porous carbon (Co-GPC) was prepared successfully using direct pyrolysis at high temperatures on a large scale (~50 g per synthesis). The as-prepared Co-GPC exhibited a typical 2D morphology and a high specific surface area of 257.8 m^2^ g^−1^. Used as supercapacitor electrode, the Co-GPC exhibited a high electrochemical capacitance of 455 F g^−1^ at a specific current of 0.5 A g^−1^. Moreover, the Co-GPC showed a stable cycling performance, with a 6.45% increase in the specific capacitance during the 2000 charge-discharge cycles at a current density of 1 A g^−1^.

## 2. Experimental Section

### 2.1. Materials

Natural flake graphite, carbon disulfide (500 mL, AR), 1-dodecanethiol (5 G, >95%), 1,3-diaminopropane (5 G, >98%), aliquot 336 (25 mL, 97%), cobalt chloride (500 g, ≥98%), 5,10,15,20-tetra(4-pyridyl)porphyrin (TPyP) (1 G, 97%), *N*-hydroxy-succinimide (NHS) (500 G, 98%), 1-(3-dimethylaminopropyl)-3-ethylcarbodiimide hydrochloride (EDC·HCl) (500 g, 98.0%), polyvinyl alcohol (PVA) (500 g, Degree of alcoholysis 98~99%), 2,2’-azobis(2-methylpropio-nitrile) (AIBN) (100 g, 99%), 2-(dodecylthiocarbonothioylthio)-2-methylpropionic acid (DDAT) (1 G, 98% HPLC), and 1,4-divinylbenzene (DVB) (250 mL, technical grade, 80%) were purchased from Aladdin (Shanghai, China). In addition, 4-vinylbenzyl chloride (VBC) (100 mL, 90%) was purchased from TCI (Tokyo, Japan), and 1,2-dichloroethane (DCE) (500 mL, AR), tetrahydrofuran (THF) (500 mL, 99.9% AR), FeCl_3_ (100 g, RG), and other chemicals were purchased from Sinopharm Chemical Reagent Co. (Shanghai, China).

### 2.2. Characterizations

The morphology and microstructures were examined using scanning electron microscopy (SEM, 25 kV, FEI Sirion-200, Tokyo, Japan) and transmission electron microscopy (TEM) measurements (TEM, 200 kV, JEOL-2100, Tokyo, Japan). Powder X-ray diffraction (XRD) measurements were carried out on a D/max-2200/PC (Rigaku Corporation, Tokyo, Japan), with Cu Kα λ = 1.5418 Å. Raman spectra were obtained using a SENTERRA (Bruker, Karlsruhe, Germany) instrument (532 nm line of Ar-ion laser and approximately 5 mW power). The chemical nature of the composites was characterized via X-ray photoelectron spectroscopy (XPS), with monochromatized Al Kα radiation (1486.6 eV). Thermogravimetry (TGA) was performed at a heating rate of 20 °C min^−1^ under an air atmosphere on a TA instrument (Q5000IR, TA instruments, New Castle, DE, USA). The Brunauer–Emment–Teller (BET) specific area was measured on an ASAP 2020 M/C surface area and porosimetry analyzer (Micromeritics Instrument Corporation, Norcross, GA, USA), based on N_2_ adsorption. FTIR spectra were recorded using a Spectrum 100 (Perkin Elmer, Inc., Waltham, MA, USA) spectrometer.

### 2.3. Preparation of 5,10,15,20-Tetra(4-pyridyl) Co (II) Porphyrin (Co(TPyP))

TPyP (1.00 g, 1.6 mmol) was dissolved in chloroform (300 mL) and stirred for 12 h in the presence of excess CoCl_2_·6H_2_O (1.10 g, 8.5 mmol). Then 40 mL of methanol was added with continued stirring for 16 h. A purple product was precipitated after adding 250 mL distilled water and after being left static for 1 h. Finally, the product was filtered and washed thoroughly with methanol and it was dried under a vacuum at 60 °C overnight.

### 2.4. Preparation of GO-PVD

As in previous work, GO nanosheets were functionalized with an amine group under the mutual action of NHS, EDC·HCl, and 1,3-diaminopropane. The as-prepared GO-NH_2_ was then esterified with acylcholoride-DDAT to form GO-DDAT. In a mixture of a PVA (0.38 g) and NaCl (0.17 g) aqueous solution (50 mL), the organic phase consisting of GO-DDAT (600 mg), VBC (6.37 g, 41.7 mmol), DVB (0.16 g, 1.2 mmol), and AIBN (33 mg) was added and stirred into the aqueous solution at 80 °C under an N_2_ atmosphere. After 8 h, the crude product was washed and filtered thoroughly with distilled water, methanol, and diethyl ether, respectively. After drying under a vacuum at 60 °C for 12 h, the pure GO-PVD was obtained.

### 2.5. Preparation of Co-GHCP and GHCP

Subsequently, the GO-PVD (1 g) and Co (TPyP) (180 mg) were dissolved in 50 mL DMF (*N*,*N*-dimethylformamide) and then ultrasonicated for 1 h to form a homogeneous suspension. The mixture was stirred at 110 °C for 3 days. After adding 20 mL DMF solution of FeCl_3_ (1.5 g), the mixture was stirred for 2 h in an ice bath. Then, the mixture was heated to 80 °C for 12 h. Finally, the product was filtered and washed thoroughly with DMF, methanol, a mixture of acetone and HCl (0.5 M), and deionized water, respectively. Co-GHCP was obtained by drying under a vacuum at 60 °C for 12 h. As a control, TPyP without cobalt ions was used as a monomer for the preparation of graphene-coupled hypercrosslinked polymer, according to the above procedure.

### 2.6. Preparation of Co-GPC and GPC

The Co-GHCP and GHCP were pyrolyzed at 800 °C for 2h under N_2_ with a heating rate of 5 °C min^−1^. After being cooled to room temperature and washed thoroughly with acetone, HCl (0.5 M), and deionized water, respectively, the porous carbons of Co-GPC and GPC were directly obtained.

### 2.7. Electrochemical Measurements

The electrical conductivity of all the products was tested using a CHI 760E workstation with a three-electrode configuration and a battery cycler (LAND-CT2001A, Btrbts, Wuhan, China) in 1.0 M KOH. For the three-electrode configuration, as-prepared samples (weight: 0.8 mg; area: 1 × 1 cm^2^) were used as a working electrode, and Pt wire and Ag/AgCl (sat. KCl) were used as the counter electrode and the reference electrode, respectively. The working electrodes were prepared as described in reference [33]. The as-prepared materials, carbon black, and polytetrafluoroethylene (PTFE), with a weight ratio of 80:10:10, were mixed thoroughly in 1.0 mL of ethanol. After drying at 80 °C for 1 h, the slurry was pressed onto a Ni foam of area ~1 × 1 cm^2^ and then vacuum dried at 60 °C for 24 h. Cyclic voltammetry (CV) and galvanostatic charge/discharge curves were carried out to test the electrochemical performance of the as-prepared electrode materials using a CHI 760E workstation. Cycling stability measurements were performed using a LAND battery electrochemical test CT 2001A. The power density (P, W kg^−1^) and energy density (E, Wh kg^−1^) were calculated as follows:

*E* = 1/2 × *C*_m_ × (Δ*V*)^2^ (1)

*P* = *E*/Δ*t* (2)

where *C*_m_, Δ*V*, and Δ*t* represent the specific capacitance (F g^−1^), discharge potential rage (V), and discharge time (s), respectively.

## 3. Results and Discussion

**Scheme 1:**
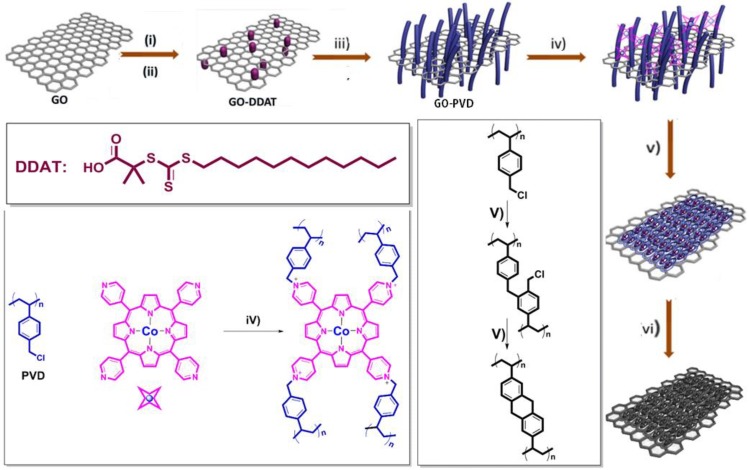
Synthesis route of cobalt-doped porous carbon nanosheets (Co-GPC): (i) *N*-hydroxy-succinimide (NHS), 1-(3-dimethylaminopropyl)-3-ethylcarbodiimide hydrochloride (EDC·HCl), 1,3-diaminopropane, water, 0 °C, 12 h; (ii) N_2_, acyl chloride-S-1-dodecyl-S’-(α,α’-dimethyl-α’’-acetic acid) trithiocarbonate (DDAT), dry dimethylformamide, Et_3_N, 0 °C, 24 h; (iii) N_2_, water, polyvinyl alcohol (PVA), NaCl, AIBN, 1,4-divinylbenzene, 4-vinylbenzyl chloride, 80 °C, 8 h; (iv) GO-PVD, Co(TPyP), dry dimethylformamide, 110 °C, 3 d; (v) FeCl_3_, 2 h; (vi) 800 °C, N_2_, 2 h.

**Figure 1:**
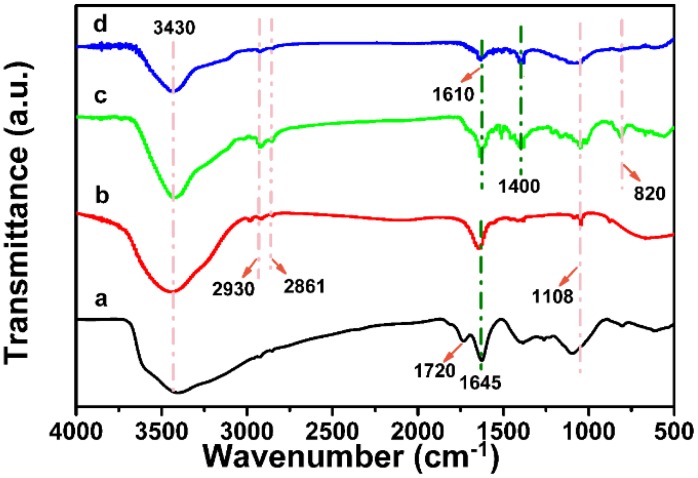
FTIR spectra of (a) GO, (b) GO-DDAT, (c) Co-GHCP, and (d) Co-GPC.

The entire synthetic route of Co-GPC is illustrated in Scheme 1. Firstly, the GO was functionalized sequentially by amine groups and 2-(dodecylthiocarbonothioylthio)-2-methylpropionic acid (DDAT) to obtain a macromolecular initiator of GO-DDAT. As shown in Figure 1a, the broad band at 3430 cm^−1^ was attributed to the O–H stretching vibrations of the GO sheets, originating from carboxylic acid and hydroxyl groups, as well as the adsorbed water [34]. The two peaks at 1720 and 1645 cm^−1^ corresponded to C=O and C=C stretching vibrations of the GO, respectively [35]. For the GO-DDAT (Figure 1b), the peak of the O-H stretching vibrations was suppressed, whilst the hydrogen bond interactions at 1108 cm^−1^ were observed clearly, suggesting the existence of an amido linkage esterified from the carboxylic group. Furthermore, the absorption bands at 2930 and 2861 cm^−1^ were ascribed to the methylene and methyl stretching of DDAT, indicating the successful grafting of the chain-transfer agent of DDAT onto the surface of the GO [33]. The grafting ratio of DDAT in GO-DDAT calculated using elemental analysis reached 3.3%. Used as the RAFT macromolecular chain-transfer agent of GO-DDAT, monomers of 1,4-divinylbenzene and 4-vinylbenzyl could be grafted onto the GO surface through traditional RAFT emulsion polymerization in water, with the initiator of AIBN and emulsifier of PVA. Owing to the existence of methyl chloride, 5,10,15,20-tetra(4-pyridyl) Co (II) porphyrin (Co (TPyP)) could be crosslinked onto the GO-PVD through the nucleophilic substitution reaction to form Co-GO-PVD. The 2D porous hypercrosslinked polymer of Co-GHCP was then prepared using a FeCl_3_-catalyzed Friedel-Crafts reaction. Compared to that of GO-DDAT, Co-GHCP exhibited a significant peak at 1610 cm^−1^ (skeletal vibrations from the graphitic domains), attributed to the aromatic C=C. The vibration peaks at ~1400 and ~1000 cm^−1^ could be attributed to the –Co–N– bonds of the porphyrin rings [36]. Additionally, a distinctive peak at 820 cm^−1^ corresponding to C–Cl stretching was detected, which suggested a low reaction activity for the Friedel-Crafts reaction in the hypercrosslinked polymer, due to the restriction motion of the polymeric chains (Figure 1c) [37]. After the heat treatment, the disappearance of the peak at 820 cm^−1^ in the Co-GPC indicated the complete transformation of the unreacted Ar–CH_2_–Cl groups for the graphene-like carbon structure. More importantly, the typical peaks of the porphyrin group still existed (Figure 1d). All these results confirmed the successful doping of cobalt ion onto the skeleton of the graphene-coupled hypercrosslinked polymer and corresponding porous carbon. In the control experiments, GHCP and GPC were also achieved by using TPyP instead of Co (TPyP), according to the same procedure.

The thermal behaviors of Co-doped Co-GHCP and un-doped GHCP were recorded using thermal gravimetric analysis (TGA) at a heating rate of 20 °C min^−1^ under a nitrogen atmosphere. Thermogravimetric (TGA) curves of the Co-GHCP and GHCP were recorded in Figure S1, where the weight loss (<10%) below 250 °C was attributed to the evaporation of adsorbed small molecules and crystal water. For the GHCP, only 8% weight loss was achieved at a temperature of 350 °C. Rapid weight loss occurred in the range between 400 to 500 °C, indicating the decomposition of the alkane structure in the main chain of the hypercrosslinked polymer and some oxygen-containing groups in the GO. These results demonstrated that the graphene-coupled hypercrosslinked polymer exhibited high thermal stability. After 500 °C, the residual weight of the GHCP was almost constant, which suggested that the samples had been converted to stable carbon. The residual weight of the GHCP at 800 °C was 28.2 wt%. Co-GHCP showed a similar decomposition temperature, reaching to 400 °C. However, the weight loss continued when the temperature was over 450 °C, and the residual weight was 18.4 wt% at 800 °C. This phenomenon could be attributed to the decomposition of the Co (II) porphyrin group, where the dissociation of cobalt ion with the generation of radical groups may have induced the decomposition of the porphyrin groups [38]. Overall, the Co-GHCP and GHCP had excellent thermal stability and moderate carbon yields, which were good precursors for the preparation of carbon materials (PCs).

**Figure 2:**
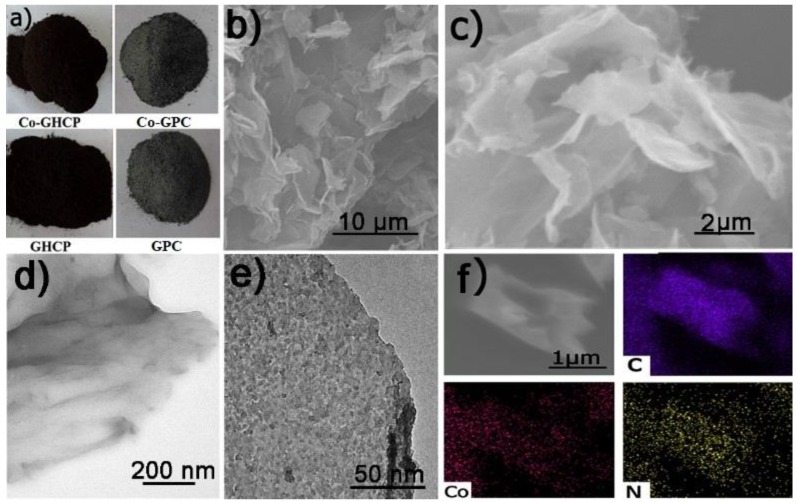
Morphology characterization of as-prepared nanosheets. (**a**) Co-GHCP, GHCP, Co-GPC and GPC. Scanning electron microscopy (SEM) (**b**,**c**), transmission electron microscopy (TEM) (**d**,**e**), and SEM-EDX (Energy Dispersive X-Ray Spectroscopy) elemental mapping (**f**) of Co-GPC.

As-prepared Co-GHCP and GHCP showed solid powder with the color of atropurpureus. After the heat treatment, the color of the Co-GPC and GPC changed to black (Figure 2a). As a typical example, the morphology and microstructure of the porous carbon Co-GPC was investigated using scanning electron microscopy (SEM) and transmission electron microscopy (TEM). In Figure 2b–e, the Co-GPC showed several free-standing nanosheets, with a size ranging from a hundred nanometers to several micrometers, which was similar to other graphene-coupled porous polymers and porous carbon. In addition, the 2D morphology with visible wrinkles could be clearly observed even after high temperature pyrolysis, indicating the flexibility feature of the porous carbon nanosheets, originating from the template of graphene [39]. More importantly, no bare graphene sheets or free porous polymeric nanoparticles were observed in either the SEM or TEM, which was attributed to the efficient RAFT polymerization and nucleophilic substitution reaction for the polymerization of monomers on the surface of the GO. In the enlarged TEM image, alternating light and dark regions could be easily observed, suggesting the creation of a porous structure in the framework of carbon materials during pyrolysis [40]. As indicated by elemental mapping in Figure 2f, homogeneous distributions of C, N, and Co elements in the Co-GPC further confirmed the introduction of the Co (II) porphyrin group into the skeletal framework of the porous carbon.

**Figure 3:**
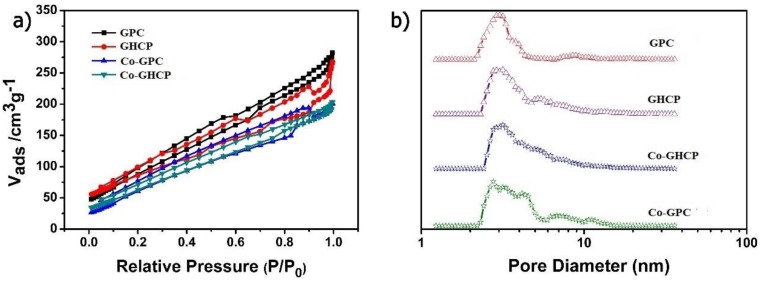
(**a**) Nitrogen adsorption/desorption isotherm plot and (**b**) the Barrett–Joyner–Halenda (BJH) pore-size distribution curve of GHCP, GPC, Co-GHCP, and CPC.

To reveal the porous nature of the as-prepared materials, the nitrogen adsorption/desorption properties of the Co-GPC, Co-GHCP, GPC, and GHCP were tested. As shown in Figure 3a, the hysteresis loops of the isotherms for all the samples could be classified as type IV, according to the International Union of Pure and Applied Chemistry (IUPAC) classification, indicating the presence of many mesopores in the porous materials [41]. The specific surface areas of the Co-GHCP and GHCP were 130.8 and 143.7 m^2^ g^−1^, respectively, based on Brunauer–Emmett–Teller (BET) calculations. These small specific surface areas were ascribed to the large number of flexible polymeric chains, as well as the chloride anions in the hypercrosslinked polymeric frameworks. Moreover, the pore size distributions of the Co-GHCP and GHCP analysis using nonlocal density functional theory (NL-DFT) revealed similar mesopores centered around 3.0 nm (Figure 3b). After pyrolysis at 800 °C, the BET surface areas of the porous carbon nanosheets were increased significantly relative to the pristine hypercrosslinked polymers. In this study, the specific surface areas of the Co-GPC and GPC were found to be 257.8 and 272.5 m^2^ g^−1^, respectively. Notably, the average pore sizes of the porous carbon materials had also relatively increased, which showed several broad peaks between 3 and 7 nm. This possibly alludes to an increase of the rigid framework through the partial degradation of the polymer chain and recombination of the fragments under the carbonization conditions, as described in reference [31]. The porous data for the as-prepared samples is listed in Table 1.

**Figure 4:**
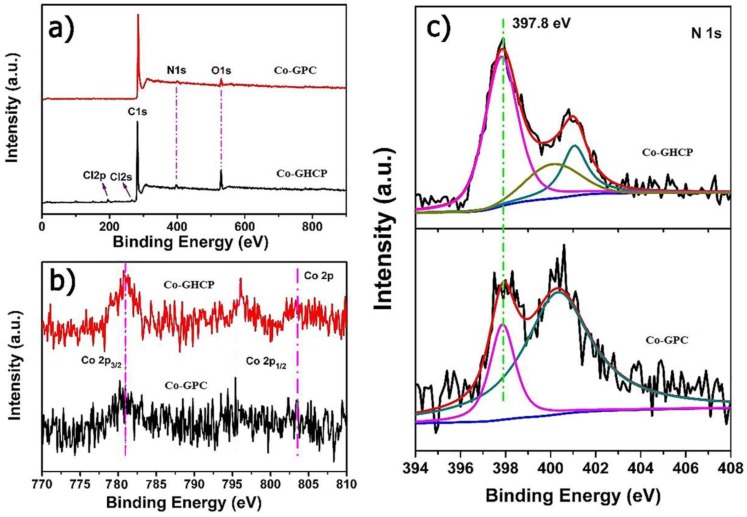
X-ray photoelectron spectroscopy of Co-GHCP and Co-GPC (**a**) survey, (**b**) Co 2p, (**c**) N 1s, respectively.

X-ray photoelectron spectroscopy (XPS) was carried out to elucidate the valence states of the individual elements of Co-GHCP and Co-GPC. The typical XPS survey spectra for Co-GHCP and Co-GPC are illustrated in Figure 4a. For Co-GHCP, the peak at 531 eV corresponded to the O 1s attributed to the hydroxyl and carbonyl group on the surface of the GO. Meanwhile, the signals of methyl chloride were also observed, which was consistent with those of the FTIR results. During the carbonization process, both the oxygen and chloride peaks were significantly suppressed. The high-resolution spectra of Co and N are given in Figure 4b,c. The Co in the Co-GHCP was comprised of two main peaks, where the peaks at about 782.1 eV (2p_3/2_) and 804.1 eV (2p_1/2_) were assigned to the Co (II) of the cobalt porphyrin blocks [36]. Furthermore, a peak at 397.8 eV in the high-resolution N 1s spectra of pristine Co-GHCP was characteristic of phthalocyanines, which reflected the configuration of the Co-N_4_ center as a mesomeric bridging of the cobalt ion with ideally four equivalent nitrogens. In the N 1s spectra of Co-GHCP, the peaks at 400.1 eV and 401.1 eV could be assigned to pyrrole N-Co and quaternary N, which indicated the successful grafting of 5,10,15,20-tetra(4-pyridyl) Co (II) porphyrin onto the GO-PVD [41]. After thermal treatment, the peaks at 782.1 eV and 397.8 eV in the Co 2p and N 1s spectra of the Co-GHCP, respectively, were seen clearly, further confirming that most of the cobalt was presented as a complex state of the Co (TPyP) structure. These results indicated the successful incorporation of Co and N into the porous carbon of the Co-GPC microstructures.

**Figure 5:**
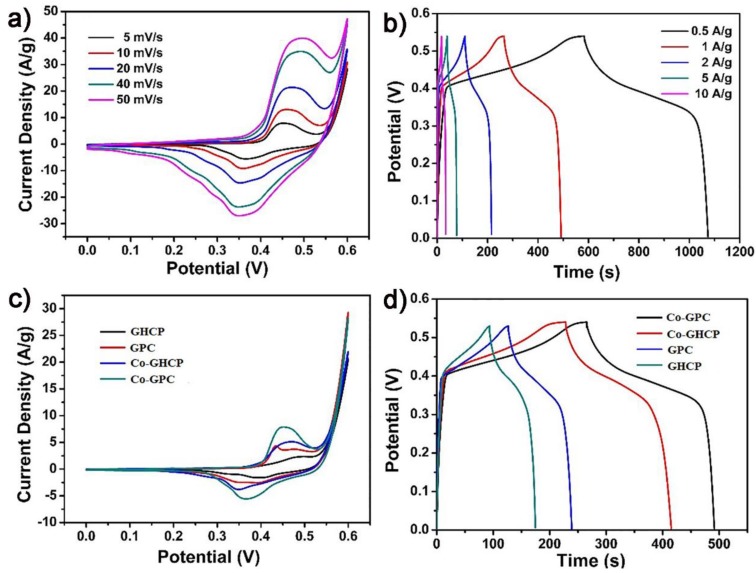
(**a**) Cyclic voltammograms (CV) of the Co-GPC electrodes at various scan rates; (**b**) The galvanostatic charge/discharge curves of the Co-GPC supercapacitor at various specific currents; (**c**) CV of Co-GHCP, Co-GPC, GHCP, and GPC at 10 mV s^−1^; (**d**) The charge/discharge curves of the Co-GHCP, Co-GPC, GHCP, and GPC performed at the current density of 1 A g^−1.^

The unique geometric structures, featuring heteroatom doping, 2D morphology, as well as the moderate surface area of graphene-coupled hypercrosslinked polymers and corresponding porous carbons may serve as promising candidates for electrochemical capacitive applications. The supercapacitor performance of the as-prepared materials as working electrodes was measured using a standard three-electrode system, with platinum wire as the control electrode and Ag/AgCl as the reference electrode under alkaline conditions (1 M KOH). As illustrated in Figure 5a, all the cyclic voltammetry (CV) curves of Co-GPC at various scan rates from 5 mV s^−1^ to 50 mV s^−1^ exhibited rectangular-like shapes with distinct redox peaks during the charging/discharging process between 0.15–0.55 V. These symmetrical and wide redox peaks were consistent with the faradic peaks in the CV experiment, demonstrating the excellent and reversible pseudocapacitor status of the Co-GPC. With an increasing scan rate, the current density of the redox peaks increased linearly with similar shapes of the CV curves, indicating a fast and highly stable redox reaction between the electrode and the electrolyte during the charge/discharge process for the electrodes. Meanwhile, its galvanostatic charge/discharge curves at various galvanostatic specific currents of 0.5, 1, 2, 5, and 10 A g^−1^ at the potential range of 0–0.54 V exhibited approximately symmetric shapes with small plateaus (Figure 5b) [42]. When the current density increased, the charge/discharge time was significantly shortened. Compared to those of the Co-GPC, the similar redox peaks of the CV curves were also observed in Co-GHCP, GHCP, and GPC (Figure S2). These results could be ascribed to the introduction of the porphyrin unit with a high redox property, based on previous work [33]. However, the potentials of the redox peaks for Co-GHCP and Co-GPC were broader than those of GHCP and GPC under the same scan rate of 10 mV s^−1^ (Figure 5c), which may be attributed to the incorporation of a metal complex with a high redox behavior [43]. At the same current density of 1 A g^−1^, the charge/discharge time gradually increased for the sequence of GHCP, GPC, Co-GHCP, and Co-GPC (Figure 5d), which was consistent with the above CV results.

**Figure 6:**
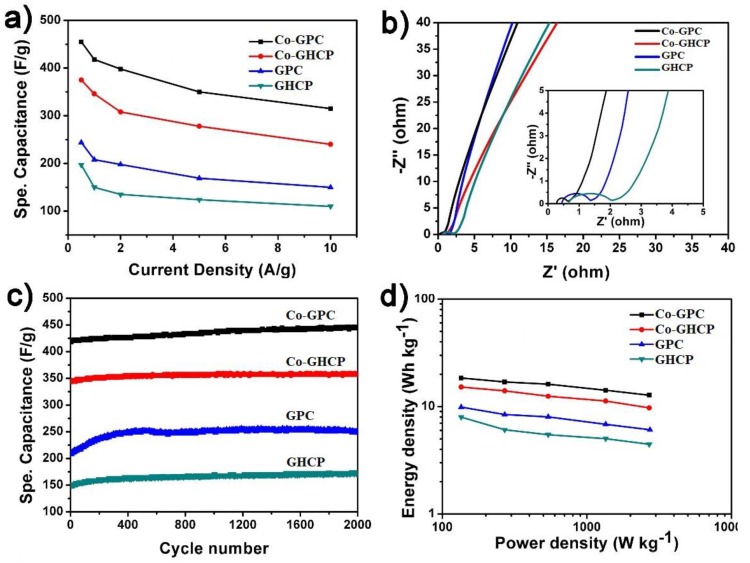
(**a**) Specific capacitance of the Co-GHCP, Co-GPC, GHCP, and GPC. (**b**) Electrochemical impedance spectroscopy (EIS) plots of the Co-GHCP, Co-GPC, GHCP and GPC, Inset: the low frequency region; (**c**) Cycling performance of the Co-GHCP, Co-GPC, GHCP, and GPC at the specific currents of 1 A g^−1^; (**d**) Ragone plot of the Co-GHCP, Co-GPC, GHCP, and GPC.

The specific capacitance of the four samples with various specific currents is shown in Figure 6a. Calculated from the discharge parts of the galvanostatic results (Figure S3), the maximum capacitance of the GHCP, GPC, Co-GHCP, and Co-GPC was able to reach 197, 244, 375, and 455 F g^−1^, respectively, at a current density of 0.5 A g^−1^. The best capacitance performance of the Co-GPC was attributed to its low internal resistance and the excellent pseudocapacitive behavior of the Co (II) porphyrin. Accordingly, the Co-GPC was calculated as 455, 418, 398, 350, and 315 F g^−1^ at different specific currents of 0.5, 1, 2, 5, and 10 A g^−1^, respectively, showing its attractive rate performance even at a high current density. To gain insight into the prominent electrocapacitance behaviors, the conductivity of the as-prepared materials was further verified via electrochemical impedance spectroscopy (EIS) analysis in 1 M KOH, with an alternating current (AC) amplitude of 5 mV (Figure 6b). The diameter of the incomplete semicircle of Co-GPC in the high frequency region was smaller than that of GHCP and GPC, indicating its lower electron transfer resistance (R_ct_) by formation of a Co–N–C bond within the carbon matrix. Moreover, the intercepts of the electrochemical impedance spectroscopy (EIS) curves for ohmic resistance (R_S_) between the current collector and the active materials indicated that the R_S_ value of the porous carbon Co-GPC and GPC was much smaller than that of the precursors Co-GHCP and GHCP, due to the higher conductivity of the carbon material, rather than that of the hypercrosslinked polymer. The circuit fitting results are show in Figure S4 and Table S1. All the results were also in agreement with that of the specific capacitance.

The cycle stability was an important issue for the supercapacitor electrode materials. The specific capacitance of the as-prepared materials was measured at a current density of 1 A g^−1^ for 2000 cycles. As shown in Figure 6c, GHCP, GPC, Co-GHCP, and Co-GPC exhibited excellent cycling stability, and the specific capacitances of the samples increased by 6.45%, which was caused by the continuous activation of electroactive material during the charge/discharge process [30]. The energy density and power density were also important parameters that were calculated to evaluate the performance of the supercapacitors. In Figure 6d, the energy densities of Co-GPC, Co-GHCP, GPC, and GHCP were obtained at 18.43, 15.2, 9.8, and 7.9 Wh kg^−1^ at a power density of 135 W kg^−1^, respectively. The maximum supercapacitor properties of the cobalt-doped porous carbon nanosheets were comparable to or even higher than many of the other porphyrin-containing porous polymers or porous carbons. More detailed comparison of the data is shown in Table S2.

## 4. Conclusions

In summary, novel 2D Co (II) porphyrin-containing hypercrosslinked polymeric nanosheets were efficiently prepared using a RAFT emulsion polymerization approach, a nucleophilic substitution reaction, and a FeCl_3_-promoted Friedel-Crafts reaction, where the nanosheets could be readily transformed into Co-doped porous carbon nanosheets using direct pyrolysis treatment. The structures and morphologies of the as-prepared materials were characterized systematically by FTIR, XPS, TEM, and SEM measurements. The resulting porous carbon showed a high specific surface area of up to 272.5 m^2^ g^−1^, which was beneficial for contact with the electrolyte, as well as for the rapid transfer of electrolyte ions. Used as electrode materials in a supercapacitor, these graphene-coupled porous carbon materials exhibited outstanding supercapacitive behavior and high stability. Notably, the specific capacitance of the Co-GPC could reach 455 F g^−1^ at a specific current of 0.5 A g^−1^. The incorporation of cobalt ion into the framework of the porous carbon not only provided a high pseudocapacitor property, but it also enhanced the conductivity of the porous material. Therefore, our approach opens a facile avenue for the preparation of metal-doped 2D porous carbon nanosheets, which could serve as promising electrochemical electrodes for a broad range of applications in electrochemical energy storage and conversion.

## Figures and Tables

**Table 1:** Nitrogen physisorption property of the prepared porous materials.

Sample	S_BET_^a^ (m^2^ g^−1^)	S_Lang_^b^ (m^2^ g^−1^)	D_av_^c^ (nm)	V_tot_^d^ (cm^3^/g)
Co-GPC	257.8	378.9	2.9	0.58
Co-GHCP	130.8	188.5	3.0	0.31
GPC	272.5	408.7	3.1	0.67
GHCP	143.7	209.8	3.3	0.34

Surface areas calculated from the N_2_ adsorption isotherm using the ^a^ Brunauer–Emmett–Teller (BET) and ^b^ Langmuir methods; ^c^ average pore size based on the adsorption isotherm; ^d^ Total pore volume at p/p_0_ = 0.99.

## Supplementary Materials

The following are available online at http://www.mdpi.com/2073-4360/10/12/1339/s1. Supplementary data of the TGA, XRD, Electrochemical results related to this article can be found at https://doi.org/10.1016/.
