# Supplementary material for: Cobalt-Doped Porous Carbon Nanosheets Derived from 2D Hypercrosslinked Polymer with CoN4 for High Performance Electrochemical Capacitors
Source: Polymers (Basel). 2018 Dec 4;10(12):1339. doi: 10.3390/polym10121339 (PMC6401960; doi:10.3390/polym10121339)
Supplement: Supplementary file 1 [file polymers-10-01339-s001.pdf]

polymers

Supplementary Materials

Cobalt-doped Porous Carbon Nanosheets Derived from 2D Hypercrosslinked Polymer with CoN_4 for High Performance Electrochemical Capacitors

Yuanhai Chen [1], Fengru Liu [1], Feng Qiu [1,2,*], Chenbao Lu [3], Jialing Kang [1], Doudou Zhao [1], Sheng Han [1,*] and Xiaodong Zhuang [3,*]

1 School of Chemical and Environmental Engineering, Shanghai Institute of Technology, Haiquan Road 100, Shanghai 201418, China; yuanhai_charles@163.com (Y.C.); lfru0719@163.com (F.L.); 176061210@mail.sit.edu.cn (J.K.); 176062121@mail.sit.edu.cn (D.Z.)
2 School of Chemistry, University of Bristol, Cantock's Close, Bristol BS8 1TS, UK
3 School of Chemistry and Chemical Engineering, State Key Laboratory of Metal Matrix Composites, Shanghai Jiao Tong University, 800 Dongchuan Road, Shanghai 200240, China; castle@sjtu.edu.cn
* Correspondence: feng.qiu@bristol.ac.uk (F.Q.); hansheng654321@sina.com (S.H.); zhuang@sjtu.edu.cn (X.Z.)

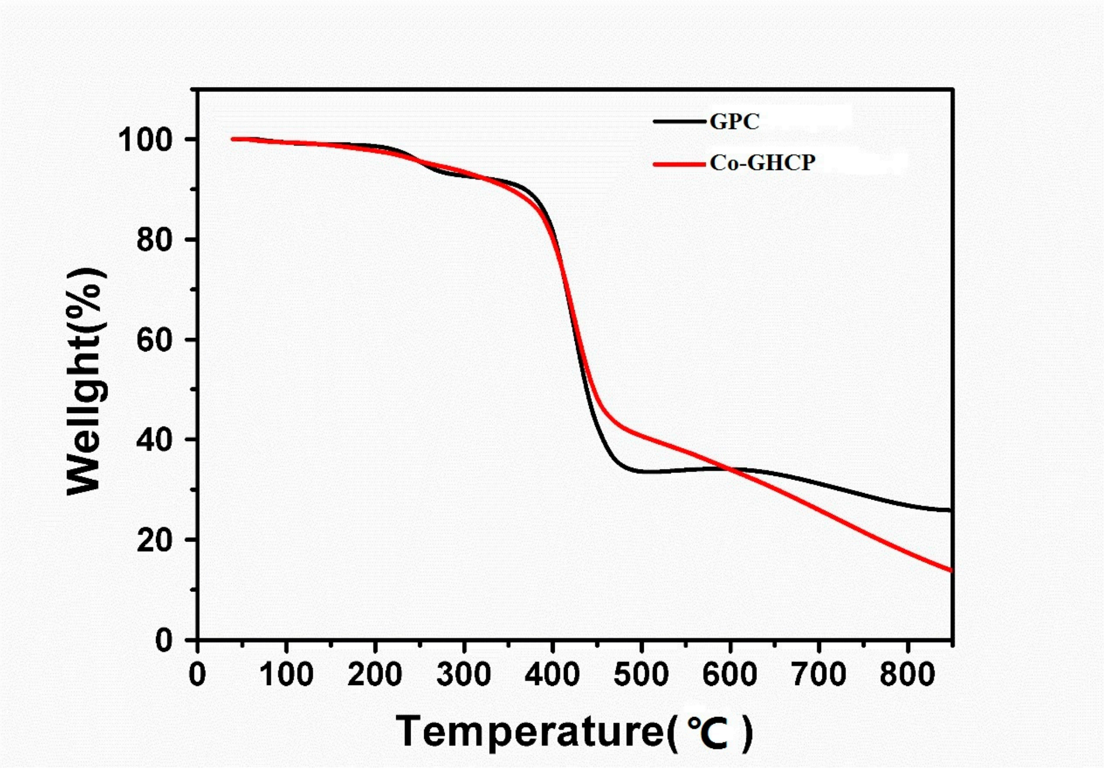

Figure S1. Thermo-gravimetric analysis (TGA) curve of Co-GHCP and GPC.

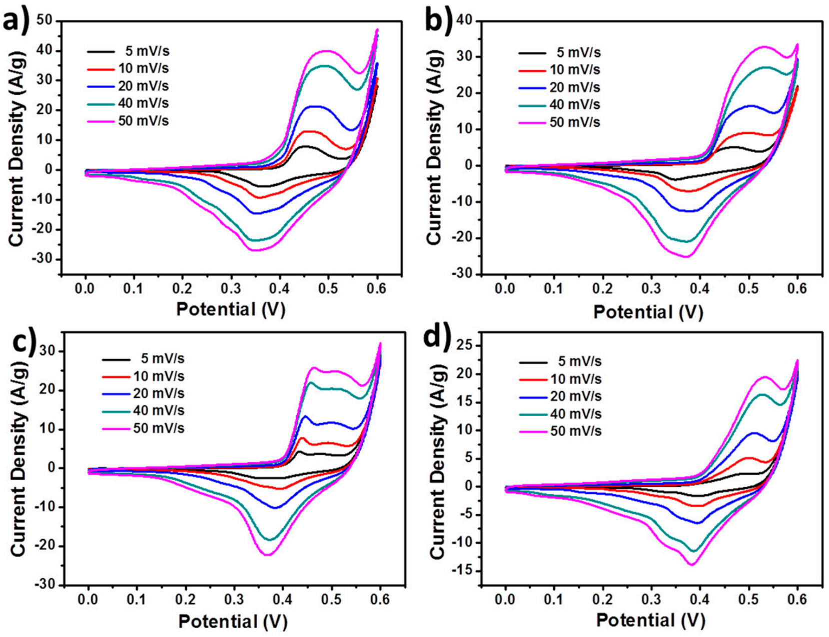

Figure S2. CV curves of (a) Co-GPC, (b) Co-GHCP, (c) GPC and (d) GHCP electrodes at various scan rates of 5, 10, 20, 40 and 50 mV s^{-1} in the range of 0~0.6 V.

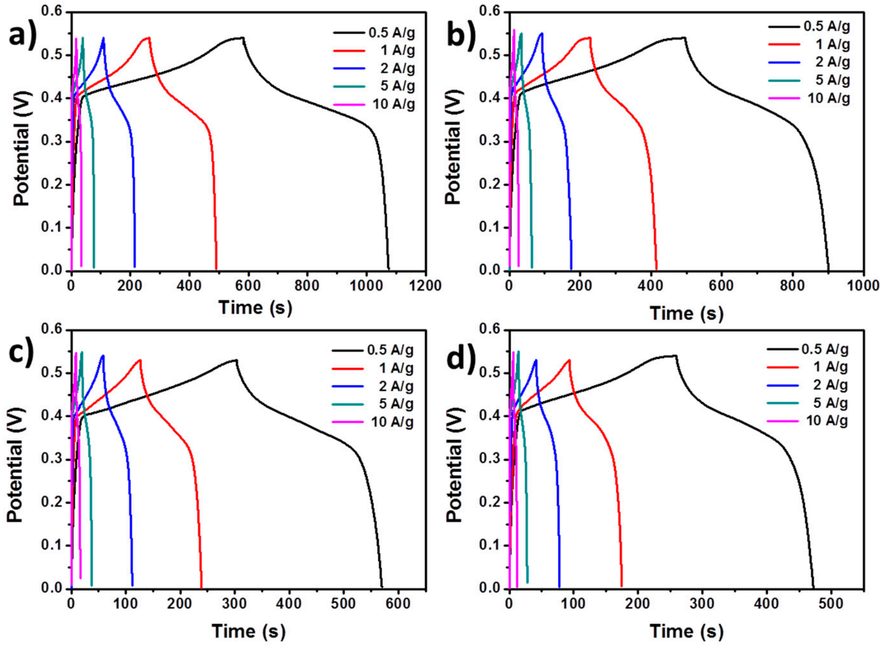

Figure S3. The galvanostatic charge-discharge curves of (a) Co-GPC, (b) Co-GHCP, (c) GPC and (d) GHC electrodes at various specific currents of 0.5, 1, 2, 5 and 10 A g^{-1} in the range of 0~0.54 V.

a)

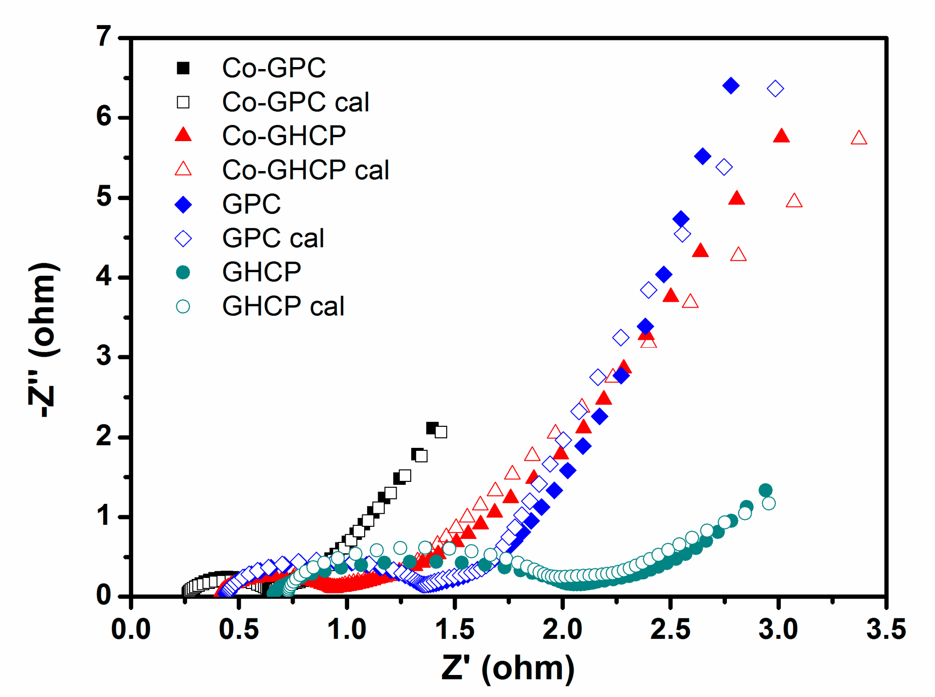

b)

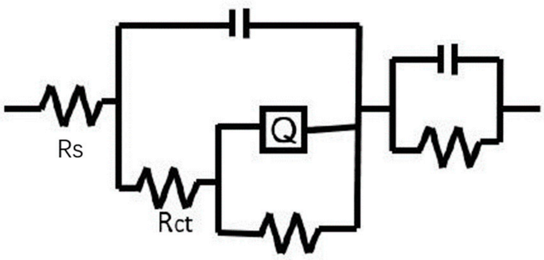

Figure S4. (a) The magnified EIS plot of the low frequency region (solid: experimental data; open: fitting data); (b) the equivalent circuit used for fitting of EIS data.

Table S1. The charge-transfer resistance values (Rct) and the solution resistance values (Rs) of the samples as electrodes of supercapacitors in 1 M KOH.

**Sample**	**Rct (Ω)**	**Rs (Ω)**
Co-GPC	0.28	0.26
Co-GHCP	0.35	0.42
GPC	0.47	0.45
GHCP	0.38	0.72

Table S2. Comparison of capacitance performance with other porphyrin-containing porous polymers or porous carbons.

Sample	Electrolyte	Maximum C_s	Rate performance	Reference
Co-doped porous carbons nanosheets from 2D hypercrosslinked polymer containing Co porphyrin	1 M KOH	455 F g^{-1} at 0.5 A g^{-1}	C_s retention of 69.2% at 10 A g^{-1}	This work
		418 F g^{-1} at 1.0 A g^{-1}	C_s retention of 75.4% at 10 A g^{-1}	
2D CoSNC nanocomposites from porphyrin-based MOF	2 M KOH	360.1 F g^{-1} at 1.5 A g^{-1}	C_s retention of 74.3% at 15.0 A g^{-1}	Ref. 1
Microporous Poly(zincporphyrin)	0.1 M Bu_4NPF_6	142 F g^{-1} at 5.0 A g^{-1}	C_s retention of ~70% at 50 A g^{-1}	Ref. 2
Cu-porphyrin@PPy nanocomposites	0.5 M H_2SO_4	~496 F g^{-1} at 1.0 A g^{-1}	C_s retention of ~57% at 10 A g^{-1}	Ref. 3
Fe-doped porous carbon from Fe porphyrin-based microporous conjugated polymer	1 M H_2SO_4	~182 F g^{-1} at 1.0 A g^{-1}	C_s retention of ~67 % at 10 A g^{-1}	Ref. 4
Nanoporous carbons from porphyrin-based MOF	1 M H_2SO_4	425 F g^{-1} at 2.0 A g^{-1}	C_s retention of ~57.4 % at 10 A g^{-1}	Ref. 5
Fe-doped porous carbons from Fe porphyrin-based coordination polymers	-	380 F g^{-1} at 1.0 A g^{-1}	C_s retention of ~52 % at 10 A g^{-1}	Ref. 6
Co-doped porous carbons from Co porphyrin-based coordination polymers	-	100 F g^{-1} at 1.0 A g^{-1}	C_s retention of ~35 % at 10 A g^{-1}	

Reference

1. F. Cao, M. Zhao, Y. Yu, B. Chen, Y. Huang, J. Yang, X. Cao, Q. Lu, X. Zhang, Z. Zhang, C. Tan, H. Zhang, Synthesis of Two-Dimensional CoS1.097/Nitrogen-Doped Carbon Nanocomposites Using Metal–Organic Framework Nanosheets as Precursors for Supercapacitor Application, Journal of the American Chemical Society, 138 (2016) 6924-6927.
2. H. Zhang, Y. Zhang, C. Gu, Y. Ma, Electropolymerized Conjugated Microporous Poly(zinc-porphyrin) Films as Potential Electrode Materials in Supercapacitors, Advanced Energy Materials, 5 (2015) 1402175.
3. H. Yao, F. Zhang, G. Zhang, H. Luo, L. Liu, M. Shen, Y. Yang, A novel two-dimensional coordination polymer-polypyrrole hybrid material as a high-performance electrode for flexible supercapacitor, Chemical Engineering Journal, 334 (2018) 2547-2557.
4. A.C. Lim, H.S. Jadhav, J.G. Seo, Electron transport shuttle mechanism via an Fe-N-C bond derived from a conjugated microporous polymer for a supercapacitor, Dalton Transactions, 47 (2018) 852-858.
5. T.H. Chang, C. Young, M.H. Lee, R.R. Salunkhe, S.M. Alshehri, T. Ahamad, M.T. Islam, K.C.W. Wu, M.S.A. Hossain, Y. Yamauchi, K.C. Ho, Synthesis of MOF-525 Derived Nanoporous Carbons with Different Particle Sizes for Supercapacitor Application, Chemistry–An Asian Journal, 12 (2017) 2857-2862.
6. S. Jin, J.P. Hill, Q. Ji, L.K. Shrestha, K. Ariga, Supercapacitive hybrid materials from the thermolysis of porous coordination nanorods based on a catechol porphyrin, Journal of Materials Chemistry A, 4 (2016) 5737-5744.
